# Change point detection for neuronal data with CUSUM and classification methods

**DOI:** 10.1186/1471-2202-14-S1-P223

**Published:** 2013-07-08

**Authors:** Lena Koepcke, Jutta Kretzberg

**Affiliations:** 1 AG Computational Neuroscience, University of Oldenburg, 26111 Oldenburg, Germany

## 

The visual cortex relies on spikes of retinal ganglion cells (RGC) as the only source of information about the visual environment. In contrast to experimenters, the nervous system generally does not have information about the exact time points of stimulus changes and needs to infer them from RGC population activity. Purpose of change point detection methods is to identify changes in the structure of a time series. Here we want to apply and compare a non-parametric method (*classification method*) and a parametric method (*CUSUM method*) for detecting strong changes in population activity referring to stimulus changes.

**Figure 1:**
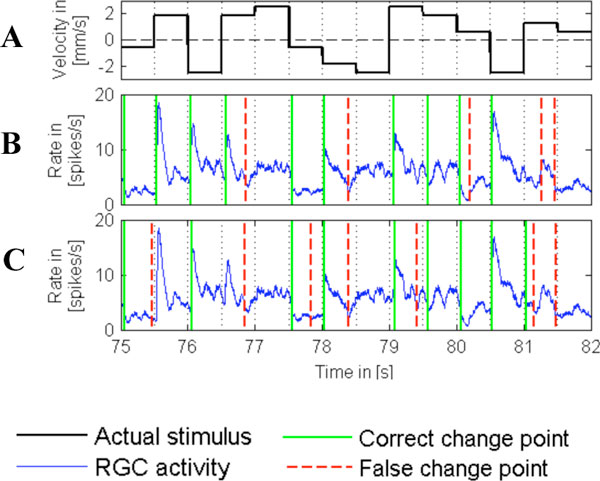
A Velocity of the pattern, (+) movement to the right, (-) to the left. B Change points detected by the classification method. CChange points detected by the CUSUM method.

The analyzed data set consists of multi-electrode recordings of RGC. The retina was stimulated with a dot pattern, which moved with one of five different speeds along one axis in two directions (Figure 1A). Since acceleration of the pattern increases and deceleration decreases neuronal activity, it is important that change point detection recognizes both types of activity changes.

*CUSUM *is a common change point detection method (e.g. [1]) with several applications in neurobiology (e.g. [2]). The basic step in CUSUM methods consists of recursive calculation of a cumulative sum. Thereby, standardized residues of an assumed parametric model are added. At a change point the value of the cumulative sum exceeds a certain threshold. Hereby, we test for each time point if the Poisson distribution has changed. The idea of the *classification method *is to use a training data set to learn the statistical properties of the population activity during constant stimulation and in response to stimulus changes. Subsequently, the learned categories are applied to the test data set to calculate for each time point the probability of a stimulus change. A change point is identified when the probability is greater than a threshold, e.g. 0.5. Comparing both methods, the advantage of the *CUSUM *method is that no training set is required and therefore less pre-analysis is needed. On the other hand, the *classification method *does not make any assumption of a parametric model. In particular, it does not assume an underlying Poisson process like *CUSUM*.

Both methods show in agreement that stimulus changes can be detected based on the population activity of RGC (Figure 1). In comparison, the *classification method *yielded better results. The fraction of correctly detected change points was slightly higher (0.8 compared to 0.7) and the fraction of false positive change points was slightly lower (0.4 compared to 0.5). Moreover, the *classification method *was found to act on shorter time scales, which might be physiologically more realistic. Considering also the more biologically realistic assumptions of the *classification method*, these results suggest that this method is superior to the established *CUSUM *method for the detection of stimulus-triggered changes in neuronal population activity.
